# Luteolin Alleviates Axitinib‐Induced Cardiomyocyte Apoptosis in Mice

**DOI:** 10.1002/jbt.71120

**Published:** 2026-10-05

**Authors:** Si‐Wen Ji, Xu Li, Dong Tang, Lin Bai, Chun‐Lei Yu, Na Niu

**Affiliations:** ^1^ Office of Academic Affairs North Sichuan Medical College Nanchong Sichuan China; ^2^ School of Pharmacy North Sichuan Medical College Nanchong Sichuan China

**Keywords:** axitinib, cardiotoxicity, luteolin, subclinical cardiac injury

## Abstract

Axitinib, a tyrosine kinase inhibitor (TKI), is associated with a risk of cardiotoxicity in clinical practice. However, its underlying toxicological mechanisms remain unclear, and effective cardioprotective strategies are lacking. This study aims to investigate the mechanisms of axitinib‐induced cardiac injury and evaluate the protective effects of luteolin. In this study, C57BL/6 mice were randomly divided into four groups: Control, Axitinib, Axitinib + Luteolin, and Luteolin alone. Ventricular systolic function (EF/FS/LVEDD/LVESD) was evaluated by echocardiography. Serum cardiac troponin T (cTnT) levels were measured to assess cardiotoxicity. Histopathological changes were observed via HE and Masson's trichrome staining. Myocardial apoptosis was quantified using TUNEL staining, and apoptosis‐related proteins (Bax/Bcl2) were analyzed by Western blot. Reactive oxygen species (ROS) accumulation was evaluated using DHE staining. The results showed that compared with the control group, axitinib did not significantly alter echocardiographic parameters but significantly elevated serum cTnT levels, indicating subclinical cardiotoxicity. Furthermore, axitinib induced notable histopathological changes, promoted aberrant ROS accumulation, upregulated the Bax/Bcl2 ratio, and triggered cardiomyocyte apoptosis. Co‐administration of luteolin significantly reduced ROS levels, restored the Bax/Bcl2 balance, and effectively alleviated axitinib‐induced cardiotoxicity and apoptosis. In conclusion, axitinib induces subclinical cardiac injury by promoting ROS generation and disrupting the Bax/Bcl2 balance, thereby activating cardiomyocyte apoptosis. Luteolin exerts significant cardioprotective effects by suppressing ROS accumulation and rectifying apoptotic pathway dysregulation, providing a promising novel strategy for the prevention and treatment of targeted therapy‐induced cardiotoxicity.

## Graphical abstract

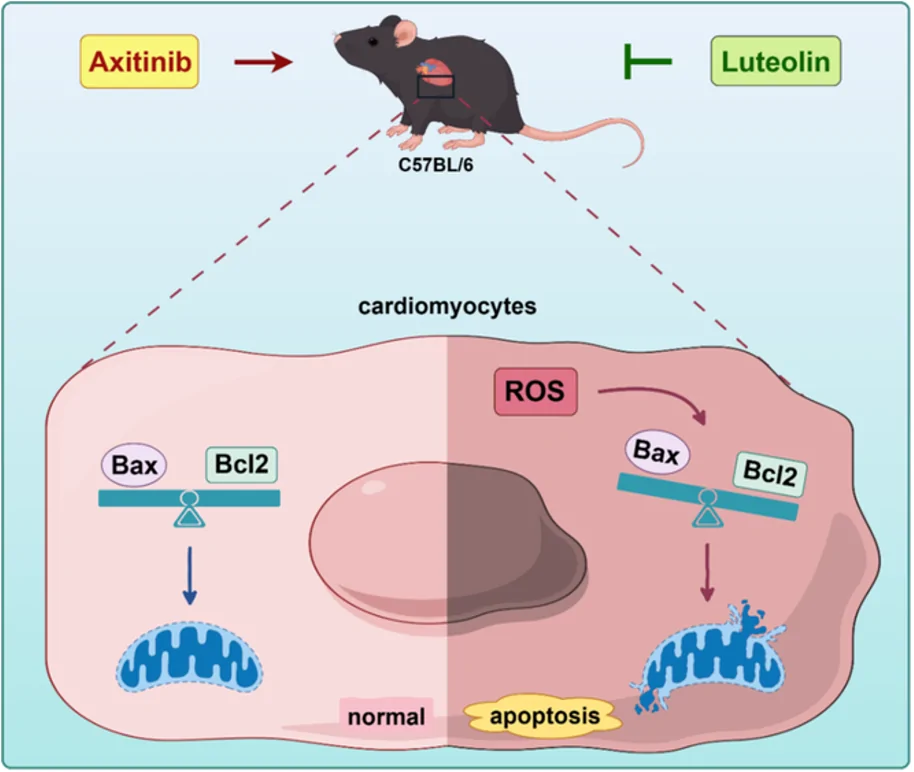

**Abbreviations**
cTnT: cardiac troponin T
DHE: dihydroethidium
LVEDD: left ventricular end‐diastolic dimension
LVEF: left ventricular ejection fraction
LVESD: left ventricular end‐systolic dimension
LVFS: left ventricular fractional shortening
TKI: tyrosine kinase inhibitor
VEGFR: Vascular endothelial growth factor receptor

## 1 Introduction

TKIs suppress cell proliferation by blocking signal transduction and are widely used in the clinical treatment of tumors and other diseases. However, pharmacovigilance data and clinical studies have shown that TKIs can induce varying degrees of cardiotoxicity, with risks varying by drug target [1, 2]. Among these inhibitors, Vascular endothelial growth factor receptor (VEGFR) inhibitors, such as axitinib, are associated with cardiovascular toxicity, which typically presents as hypertension, decreased left ventricular ejection fraction (LVEF), and heart failure [3, 4]. A clinical report revealed that a patient with metastatic renal cell carcinoma developed a decrease in LVEF and apical wall motion abnormalities 6 days after receiving axitinib combined with immunotherapy, and myocardial function gradually recovered only after drug discontinuation [5]. These findings indicate that axitinib‐related cardiotoxicity requires clinical attention, and corresponding preventive and therapeutic strategies need to be explored.

Basic research has indicated that several TKIs (such as sunitinib, imatinib, gefitinib, sorafenib, osimertinib, and crizotinib) can cause cardiotoxicity through multiple pathways. The mechanisms mainly involve mitochondrial dysfunction (such as inhibition of ATP synthesis), oxidative stress (increased ROS release), blockade of autophagy–lysosome pathways, and cytosolic Ca^2+^ overload, which then lead to myocardial cytoskeletal remodeling and apoptosis [6, 7, 8, 9, 10]. In addition, an imbalance in signaling pathways such as MAPK and PI3K is associated with the cardiotoxicity induced by drugs such as trametinib, ponatinib, and quizartinib [11, 12]. Currently, the cardiotoxic mechanisms of the aforementioned TKIs are only partially understood. However, because axitinib is a commonly used VEGFR‐TKI in clinical practice, the specific molecular mechanisms of axitinib‐induced cardiac injury remain unclear (Figure 1), which limits the development of related targeted intervention strategies.

**Figure 1:**
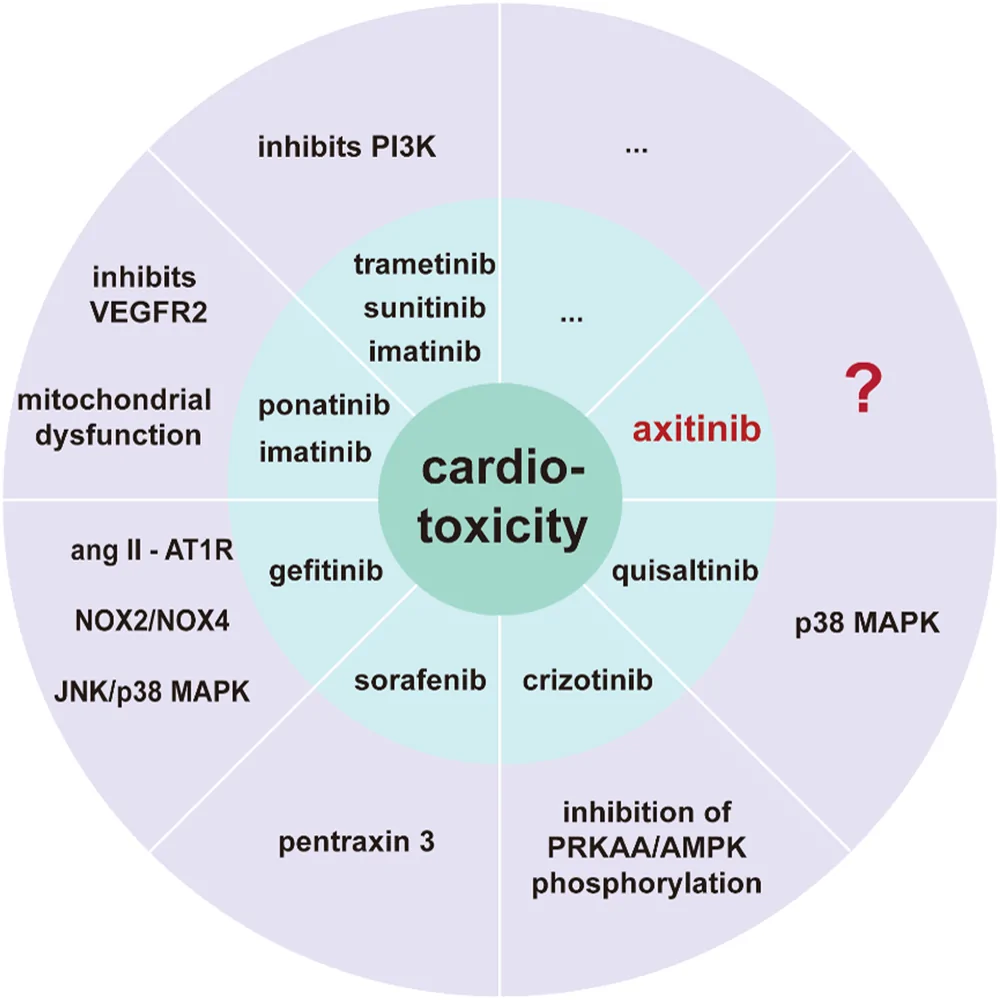
Mechanism of cardiotoxicity induced by some TKIs.

Luteolin, as a natural flavonoid compound, has been shown to have multitarget cardioprotective effects in previous studies. Studies have shown that luteolin can alleviate myocardial hypertrophy and fibrosis by inhibiting the SHP2 signaling [13], improve myocardial ischemia/reperfusion injury by activating mitochondrial BK channels [14], and exert antiapoptotic and antioxidant effects by regulating the p53 pathway [15]. Furthermore, in metabolic disorder models, luteolin can also improve cardiac dysfunction [16, 17]. On the basis of its roles in antioxidation, anti‐apoptosis, and mitochondrial regulation, luteolin may serve as a potential therapeutic agent to alleviate TKI‐induced cardiotoxicity.

Based on this background, this study aimed to investigate the effects of axitinib on the cardiac function and structure of C57BL/6 mice and its potential molecular mechanisms. We also evaluated the effects of luteolin on axitinib‐induced cardiac injury in mice to provide experimental evidence for the clinical prevention and treatment of targeted drug‐related cardiotoxicity.

## 2 Materials and Methods

### 2.1 Animals

Six‐week‐old male C57BL/6 mice (18–20 g) were purchased from GemPharmatech Co. Ltd. (China). The mice were randomly divided into four groups: the control group (intraperitoneal injection of corn oil, the solvent for axitinib; S6701, Selleck), the axitinib group (intraperitoneal injection of 50 mg/kg/day axitinib; S1005, Selleck), the axitinib + luteolin group (intraperitoneal injection of 50 mg/kg/day axitinib and intragastric administration of 100 mg/kg/day luteolin; KQUCK3KC, Picasso‐e), and the luteolin alone group. The modeling process lasted for 1 week. All mice had free access to food and water and were maintained under a 12‐h light/12‐h dark cycle. This study was approved by the North Sichuan Medical College Animal Ethics Committee (Approval No. 2024107, approved on 30 October 2024). All animal experiments were performed in strict accordance with the relevant guidelines and regulations.

### 2.2 Echocardiography

Cardiac function was evaluated using a high‐frequency ultrasound imaging system (VINNO 6 Lab X10‐23L) in mice anesthetized with isoflurane (maintained at 1.0%–1.5%). Electrocardiograms were continuously monitored, and body temperature was maintained at 37°C during the procedure. Standard views (parasternal long‐axis, short‐axis, and apical four‐chamber views) were obtained. Left ventricular systolic function was measured using M‐mode echocardiography, and diastolic function was evaluated by pulsed‐wave Doppler. All parameters were averaged over three consecutive cardiac cycles.

### 2.3 Serum cTnT Measurement

The concentration of cTnT in the serum of mice from each group was detected according to the manufacturer's instructions of the assay kit.

### 2.4 Histological Staining

Mouse heart tissues were fixed in 4% paraformaldehyde, dehydrated through a graded ethanol series, embedded in paraffin, and sectioned at a thickness of 5 μm. After deparaffinization and rehydration, the sections were stained with Hematoxylin and Eosin (HE) (hematoxylin staining, differentiation in acid‐ethanol, and eosin staining) or Masson's trichrome (nuclear staining with Weigert's iron hematoxylin, followed by Ponceau‐acid fuchsin, phosphomolybdic acid, and aniline blue for collagen). The stained sections were then dehydrated with ethanol, cleared with an environmentally friendly clearing agent, and mounted with neutral resin. Myocardial structure and collagen fiber distribution were observed under an optical microscope.

### 2.5 TUNEL Staining

Paraffin sections of heart tissues were deparaffinized, rehydrated, and washed with PBS. The sections were permeabilized with 20 μg/mL proteinase K at 37°C for 20 min, followed by PBS washing. Endogenous peroxidase activity was blocked with 3% H_2_O_2_ at room temperature for 20 min, and then washed with PBS. The sections were incubated with equilibration buffer for 10 min and then covered with a labeling mixture containing Biotin‐dUTP and TdT enzyme (incubated in the dark at 37°C for 1 h). After washing with PBS, streptavidin‐HRP was added and incubated at 37°C for 30 min. Following PBS washing, DAB was applied for color development, which was monitored and stopped under a microscope. Nuclei were counterstained with hematoxylin. The sections were then dehydrated with a graded ethanol series, cleared in xylene, and mounted with neutral resin. Under a microscope, apoptotic positive nuclei appeared brown, while negative nuclei appeared blue.

### 2.6 Western Blot

Total protein was extracted from mouse heart tissues using RIPA lysis buffer, and protein concentrations were determined via the BCA method. The samples were boiled at 100°C for 5 min for denaturation. Proteins were separated by SDS‐PAGE and transferred onto nitrocellulose membranes. The membranes were blocked with 5% non‐fat milk at room temperature for 1 h and then incubated with primary antibodies against Bax (1:5000, 50599‐2, Proteintech), Bcl2 (1:1000, 26593‐1, Proteintech), and GAPDH (1:20,000, 60004, Proteintech) overnight at 4°C. After washing three times with PBS‐T for 15 min each, the membranes were incubated with HRP‐conjugated goat anti‐rabbit IgG (1:3000, RGAR001, Proteintech) or HRP‐conjugated goat anti‐mouse IgG (1:3000, SA00001‐1, Proteintech) at room temperature for 1 h in the dark. The membranes were washed again with PBS‐T three times (15 min each), developed using ECL reagent, and analyzed with a chemiluminescence imaging system.

### 2.7 Cell Culture and Cell Viability Assay

The human cardiomyocyte cell line AC16 was cultured in DMEM supplemented with 10% fetal bovine serum and 1% penicillin/streptomycin in a humidified incubator at 37°C with 5% CO_2_. For the cell viability assay, AC16 cells in the logarithmic growth phase were seeded into 96‐well plates. After attachment, the cells were subjected to specific drug treatments according to the experimental grouping. Following the treatment, Cell Counting Kit‐8 (CCK‐8) working solution was added to each well, and the plates were incubated for an additional 1‐2 h. The absorbance (OD value) at 450 nm was measured using a microplate reader to evaluate cell viability.

### 2.8 Intracellular ROS Detection (DHE Staining)

Intracellular reactive oxygen species (ROS) levels were assessed using the dihydroethidium (DHE) fluorescent probe. After drug treatment, the cells were washed with PBS and incubated with DHE working solution in the dark at 37°C for 30 min. Following incubation, the cells were washed with PBS to remove the unbound probe. The red fluorescence intensity of the cells was observed and recorded using a fluorescence microscope.

### 2.9 Reagents

Reagents not explicitly mentioned above were purchased from Servicebio.

### 2.10 Statistical Analysis

Data are presented as the mean ± standard error of the mean (SEM). Statistical analysis was performed using GraphPad Prism 9 (GraphPad Software, La Jolla, CA, USA). Multiple comparisons were preceded by Levene's test for equality of variances; if the variances were equal (*p* > 0.05), a one‐way analysis of variance (one‐way ANOVA) was applied. All experiments were performed in at least three independent replicates.

## 3 Results

### 3.1 Axitinib and Luteolin Have no Significant Effect on Cardiac Function in C57BL/6 Mice

Baseline cardiac function in each group was evaluated using transthoracic echocardiography. The results (Figure 2) revealed no statistically significant differences in the LVEF, left ventricular fractional shortening (LVFS), left ventricular end‐diastolic dimension (LVEDD), or left ventricular end‐systolic dimension (LVESD) among the groups (all pairwise comparisons, *p* > 0.5 for each). These findings demonstrate that axitinib and luteolin did not significantly affect cardiac function in C57BL/6 mice.

**Figure 2:**
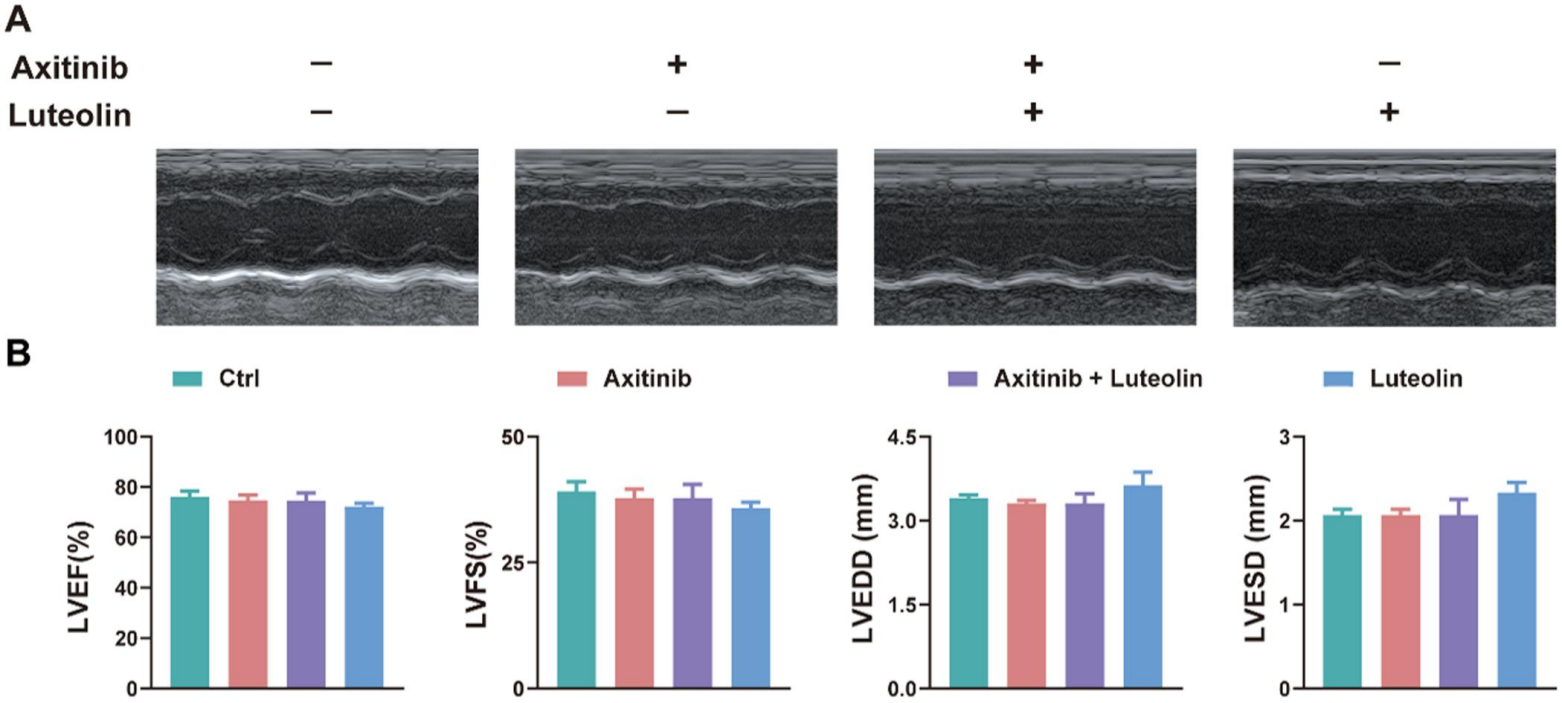
Axitinib and luteolin have no significant effect on cardiac function in C57BL/6 mice. (A) Representative echocardiographic images of mouse hearts; (B) Statistical analysis of LVEF, LVFS, LVEDD, and LVESD (*n* = 3).

### 3.2 Luteolin Reduces Axitinib‐Induced Elevation of cTnT Levels in Mice

Although echocardiography revealed no effect of axitinib on cardiac systolic function, further assessment is needed to determine potential cardiomyocyte injury. cTnT is a biomarker for cardiac injury; its elevation serves as an early warning signal that precedes functional abnormalities detectable by echocardiography [18, 19]. Serum cTnT levels in each group were measured using an assay kit. The results (Figure 3) revealed that the cTnT concentration in the axitinib‐treated group was significantly greater than that in the control group. However, compared with axitinib alone, the coadministration of axitinib and luteolin significantly decreased cTnT levels. These findings indicate that while axitinib did not impair cardiac systolic function in C57BL/6 mice, it induced significant cardiomyocyte injury (as evidenced by elevated cTnT levels), and luteolin cotreatment effectively mitigated this injury. Luteolin alone had no significant effect on the cTnT concentration. The reduction in cTnT without changes in echocardiographic parameters suggests that luteolin's mechanism likely involves cardioprotection at the cardiomyocyte level rather than direct effects on cardiac load or contractile proteins.

**Figure 3:**
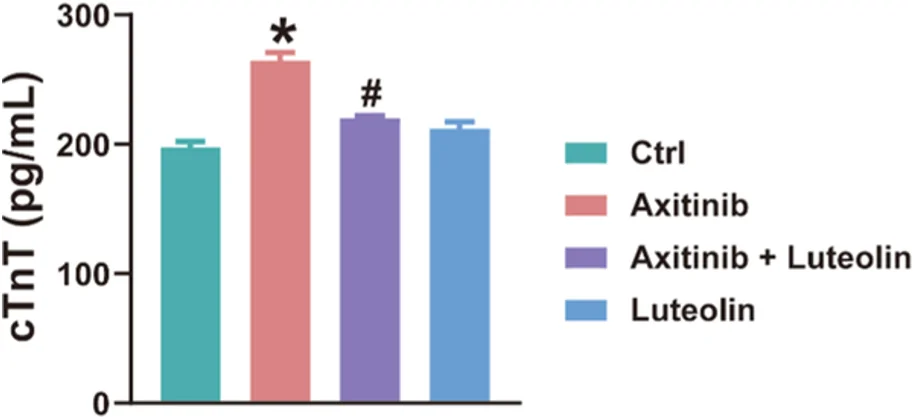
Luteolin reduces axitinib‐induced elevation of serum cTnT in mice. Serum cTnT concentration (*n* = 6, *p < 0.05 vs. control group; ^#^ *p* < 0.05 vs. axitinib group).

### 3.3 Luteolin Alleviates Axitinib‐Induced Pathological Damage in Mouse Cardiomyocytes

To further validate the pathological basis of the increase in serum cTnT levels induced by axitinib, HE staining was used to assess the degree of cardiotoxicity (inflammation/necrosis/structural abnormalities), and Masson staining was used to evaluate fibrosis. The results of the HE staining (Figure 4A) revealed the following: in the control group, the myocardial fibers were arranged in an orderly manner with normal nuclear morphology and no inflammatory infiltration or significant pathological changes. In the axitinib group, multifocal cardiotoxicity was observed and was characterized by myofibrillar disruption, inflammatory cell infiltration (indicated by arrows), and partial cardiomyocyte pyknosis (arrows). In the axitinib + luteolin group, the lesions were significantly alleviated, with only sporadic inflammatory cell infiltration and no apparent necrotic foci. Luteolin alone did not significantly differ from the control. The results of the Masson staining (Figure 4B) revealed minimal collagen deposition and disorganized myocardial fiber arrangement in the axitinib group compared with the control group. These changes were mitigated in the axitinib + luteolin group. Luteolin alone had no effect on cardiac fibrosis. These results demonstrate that axitinib induces significant pathological damage to mouse cardiomyocytes, which is attenuated by luteolin.

**Figure 4:**
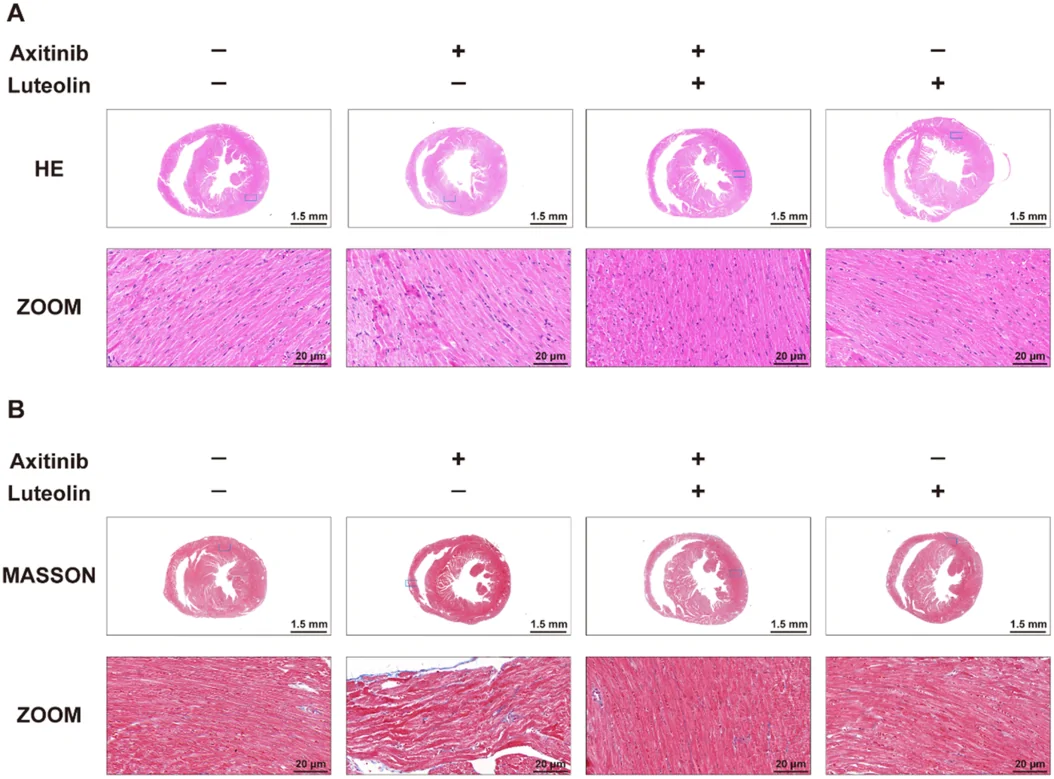
Histopathological analysis of mouse hearts by HE (A) and Masson (B) staining.

### 3.4 Luteolin Attenuates Axitinib‐Induced Cardiomyocyte Apoptosis in Mice

To further investigate the mode of cardiomyocyte death induced by axitinib, TUNEL staining was performed on cardiac tissues from each group. The results (Figure 5) revealed that compared with the control group, the axitinib group exhibited a significant increase in the number of TUNEL‐positive cells. In contrast, the axitinib + luteolin cotreatment group showed a marked reduction in the number of positive cells, whereas luteolin alone had no significant effect. This finding aligns with the ability of luteolin to reduce serum cTnT levels and alleviate histological damage. Together with the HE staining findings (nuclear pyknosis), the TUNEL staining results confirmed the occurrence of apoptotic death, demonstrating that luteolin exerts cardioprotective effects by inhibiting the apoptotic pathway. Notably, the absence of significant fibrosis on Masson staining and normal echocardiographic function indicate that axitinib‐induced cardiotoxicity remains at a reversible, apoptosis‐dominant stage, further highlighting the clinical value of early luteolin intervention.

**Figure 5:**
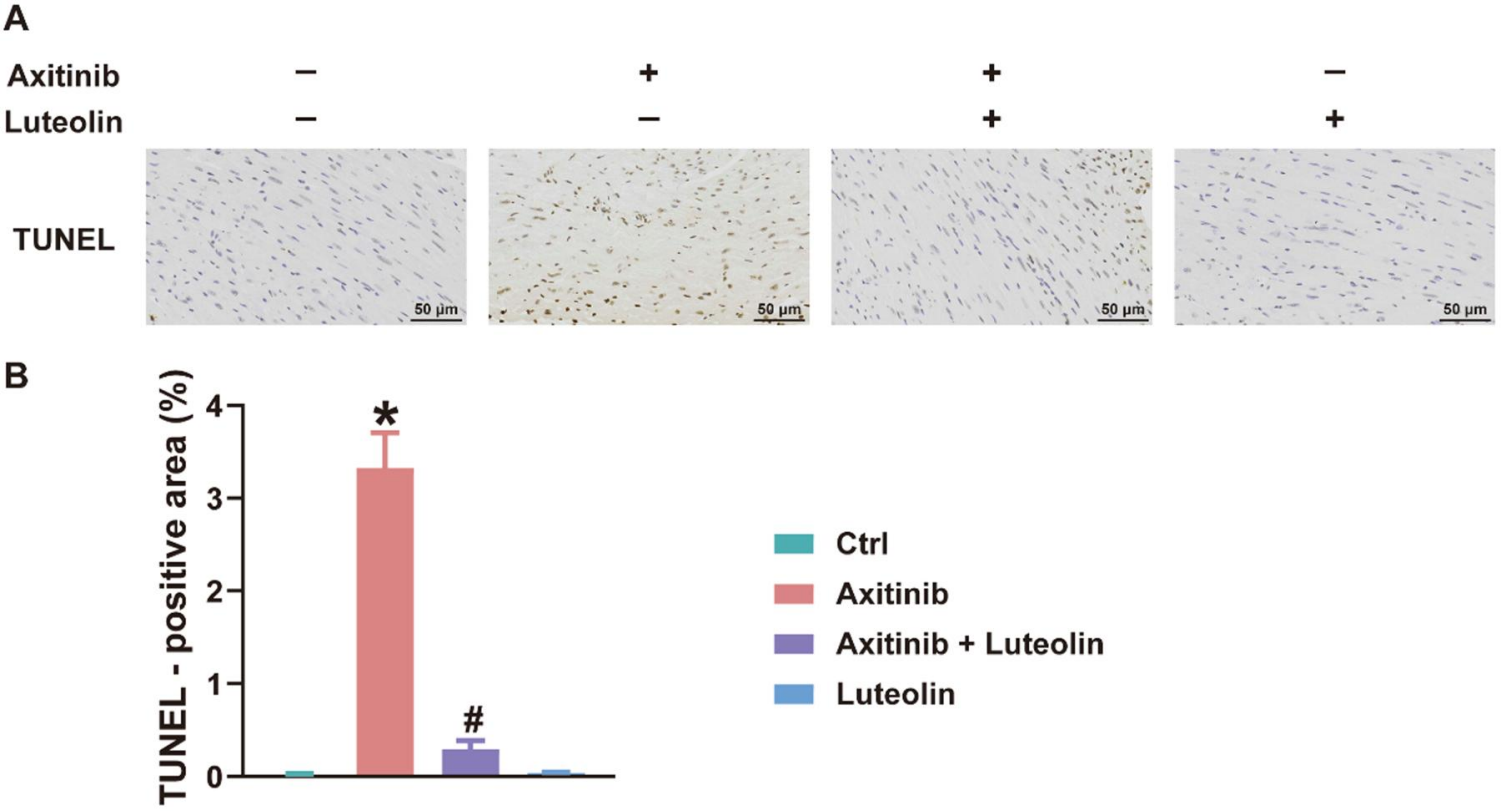
Luteolin attenuates axitinib‐induced cardiomyocyte apoptosis in mice. (A) TUNEL staining detecting apoptosis in mouse cardiac tissues; (B) Statistical analysis of apoptotic cell area proportion (*n* = 6, *p < 0.05 vs. control group; ^#^ *p* < 0.05 vs. axitinib group).

### 3.5 Luteolin Reverses the Axitinib‐Induced Cardiomyocyte Apoptosis Increase in the Bax/Bcl2 Ratio in Mouse Hearts

To investigate the mechanism of axitinib‐induced cardiomyocyte apoptosis, western blot was used to measure the protein expression levels of the key apoptosis pathway proteins Bax and Bcl2 in cardiac tissues. As shown in Figure 6, the Bax/Bcl2 ratio in the axitinib group was elevated compared with that in the control group, indicating the activation of the mitochondrial apoptosis pathway. The coadministration of luteolin effectively reversed this trend, whereas the effects of luteolin alone did not significantly differ from those of the control. These results demonstrate that axitinib induces cardiomyocyte apoptosis by increasing the Bax/Bcl2 ratio and that luteolin exerts its protective effect through this pathway.

**Figure 6:**
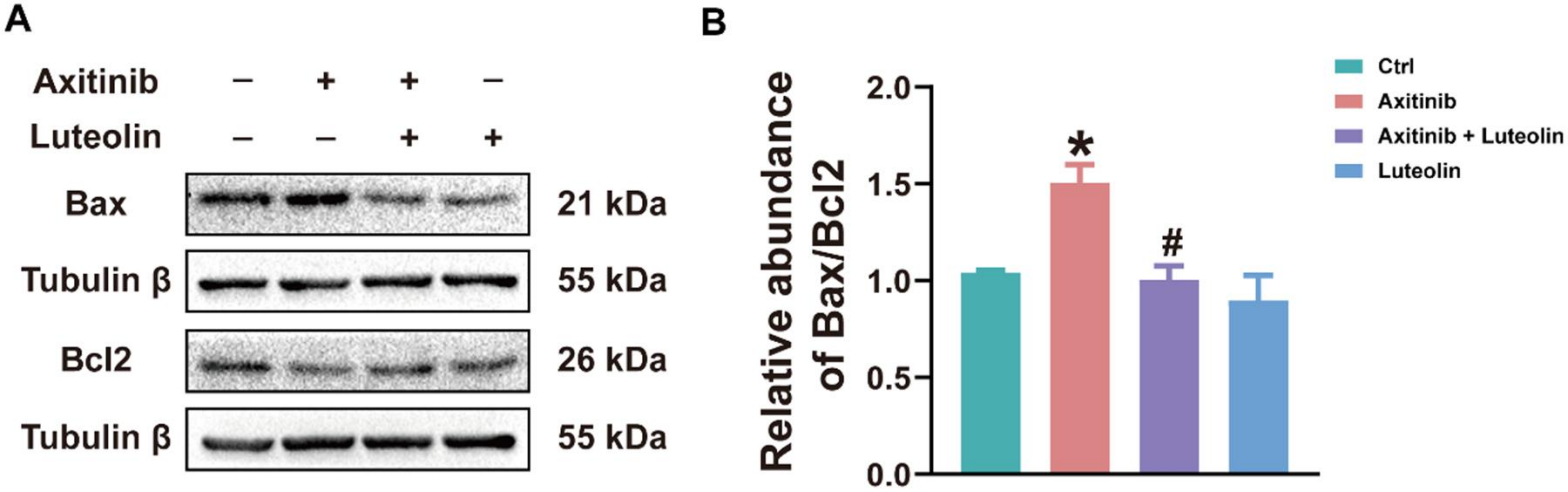
Luteolin reverses axitinib‐induced elevation of the Bax/Bcl2 ratio in mouse hearts. (A) Representative western blot images of Bax and Bcl2 protein levels in cardiac tissues; (B) Statistical analysis of Bax and Bcl2 protein expression changes (*n* = 6, *p < 0.05 vs. control group; ^#^ *p* < 0.05 vs. axitinib group).

### 3.6 Luteolin Attenuates Axitinib‐Induced Apoptosis in AC16 Cardiomyocytes via Blocking the Bax/Bcl2 Pathway and Exhibits Protective Efficacy Comparable to Captopril In Vitro

To further validate this mechanism at the cellular level, we performed in vitro experiments using the AC16 cardiomyocyte cell line. First, we evaluated the effect of axitinib on AC16 cell viability using the CCK‐8 assay, determining its half‐maximal inhibitory concentration (IC_50_) to be [10.47 μM] (Figure 7A). This provided a reliable basis for the drug concentrations used in subsequent in vitro experiments. AC16 cells were divided into four groups: ctrl, axitinib (5 μM, 24 h), axitinib + luteolin (50 μM, 24 h [20]), and luteolin. Subsequently, we performed parallel protein expression analyses in AC16 cells. The results showed that axitinib similarly induced a significant increase in the Bax/Bcl2 ratio in vitro, while luteolin treatment effectively downregulated this ratio (Figure 7B,C), which is highly consistent with our in vivo findings.

**Figure 7:**
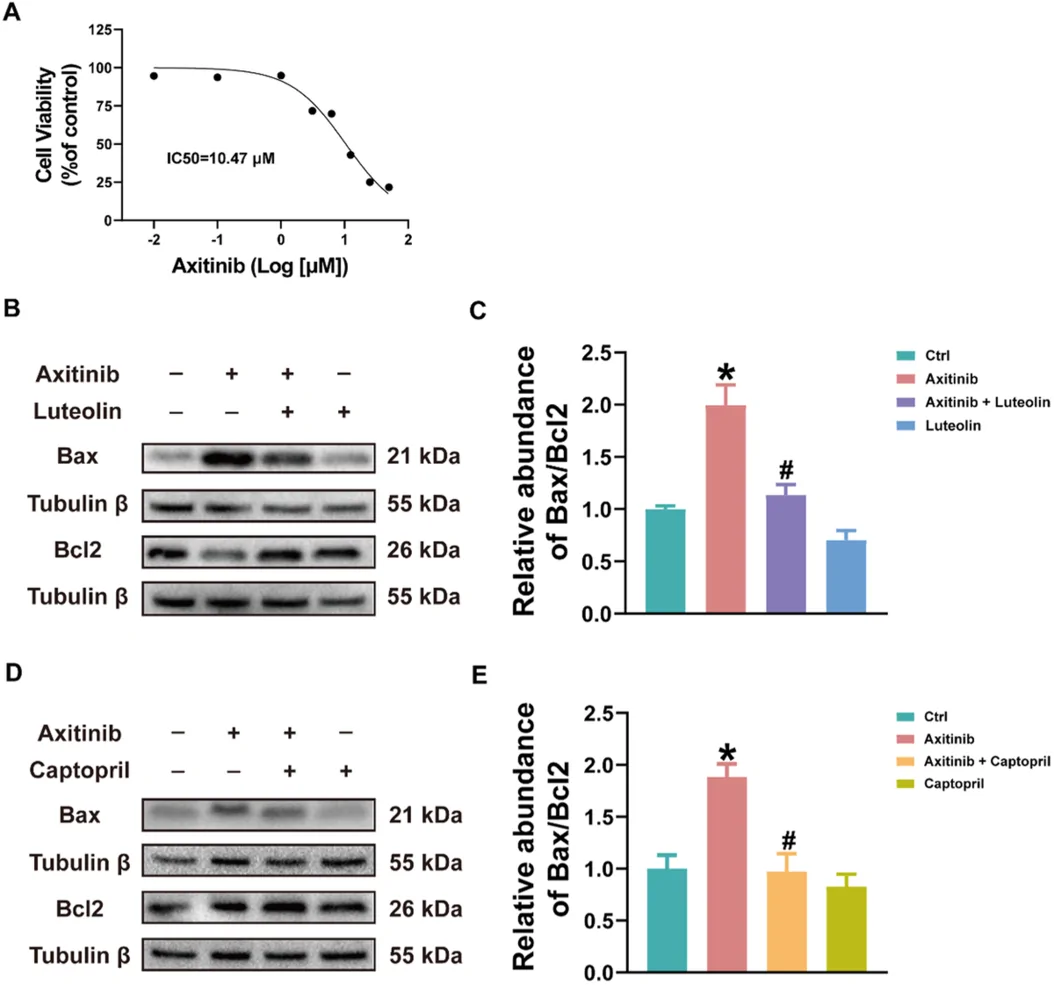
Luteolin Attenuates Axitinib‐Induced Apoptosis in AC16 Cardiomyocytes via Blocking the Bax/Bcl2 Pathway and Exhibits Protective Efficacy Comparable to Captopril In Vitro. (A) Effect of different concentrations of axitinib on AC16 cardiomyocyte viability as assessed by the CCK‐8 assay, and determination of the half‐maximal inhibitory concentration (IC_50_, *n* = 3); (B, D) Representative western blot images of Bax and Bcl2 protein levels in AC16 cells; (C, E) Statistical analysis of Bax and Bcl2 protein expression changes (*n* = 5, *p < 0.05 vs. control group; ^#^ *p* < 0.05 vs. axitinib group).

Notably, to objectively evaluate the relative protective efficacy of luteolin, we introduced captopril, a classic clinical cardiovascular drug, as a positive control in the in vitro experiments. AC16 cells were divided into four groups: ctrl, axitinib (5 μM, 24 h), axitinib + captopril (10 μM, 24 h [21]), and captopril. The results demonstrated that captopril also significantly reversed the axitinib‐induced elevation of the Bax/Bcl2 ratio (Figure 7D,E). Taken together, these in vivo and in vitro results suggest that axitinib induces cardiomyocyte apoptosis by upregulating the Bax/Bcl2 ratio, and that luteolin exerts its cardioprotective effect by blocking this apoptotic pathway, demonstrating an in vitro efficacy comparable to that of an established cardiovascular drug.

### 3.7 Luteolin Ameliorates Axitinib‐Induced Apoptosis by Inhibiting ROS Accumulation

To further explore the upstream mechanism responsible for the axitinib‐induced Bax/Bcl2 imbalance and to identify the specific intervention target of luteolin, we focused on the changes in intracellular oxidative stress (ROS) levels. As shown in Figure 8A,B, compared with the control group, axitinib significantly induced a massive accumulation of ROS in AC16 cells. However, co‐treatment with luteolin effectively suppressed this abnormal ROS burst, restoring it to a lower level. Treatment with luteolin alone had no significant effect on the basal ROS levels in the cells.

**Figure 8:**
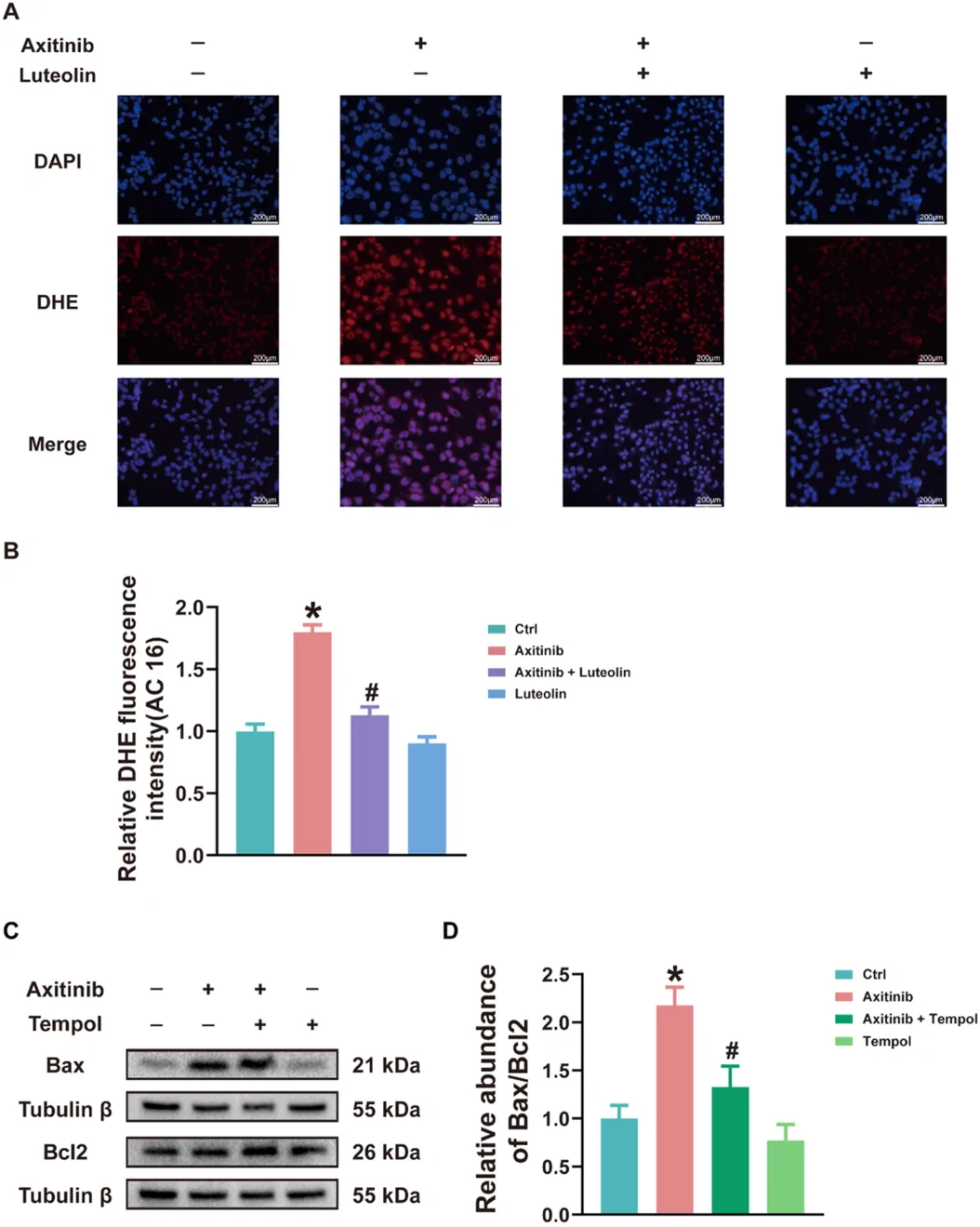
Luteolin ameliorates axitinib‐induced apoptosis by inhibiting ROS accumulation. (A) Representative DHE fluorescence images showing ROS levels in AC16 cells from each group; (B) Quantification of relative DHE fluorescence intensity in AC16 cells from each group (*n* = 6, *p < 0.05 vs. control group; ^#^ *p* < 0.05 vs. axitinib group); (C) Representative western blot images of Bax and Bcl2 protein levels in AC16 cells; (D) Statistical analysis of Bax and Bcl2 protein expression changes (*n* = 5, *p < 0.05 vs. control group; ^#^ *p* < 0.05 vs. axitinib group).

To definitively confirm whether excessive ROS accumulation is the direct cause of the downstream elevation in the Bax/Bcl2 ratio, we introduced Tempol, a well‐known broad‐spectrum ROS scavenger, for mechanistic validation in our in vitro model. AC16 cells were divided into four groups: ctrl, axitinib (5 μM, 24 h), axitinib + Tempol (250 μM, 24 h), and Tempol. The Western blot results (Figure 8C,D) demonstrated that the addition of Tempol to axitinib‐treated AC16 cells significantly reversed the axitinib‐induced elevation of the Bax/Bcl2 ratio. Treatment with Tempol alone had no obvious effect on this ratio.

Taken together, these experimental results suggest that axitinib‐induced cardiomyocyte apoptosis is highly dependent on excessive intracellular ROS production. Furthermore, a key mechanism underlying the cardioprotective effect of luteolin appears to be its antioxidant capacity to scavenge the over‐accumulated ROS, thereby blocking the downstream Bax/Bcl2‐mediated mitochondrial apoptotic signaling pathway.

## 4 Discussion

Our study revealed that axitinib induces cardiotoxicity (as evidenced by elevated cTnT concentrations and pathological changes in HE staining) by increasing ROS levels, which in turn disrupts the Bax/Bcl2 balance and leads to an increase in the number of TUNEL‐positive cardiomyocytes. Concurrently, luteolin mitigates this damage by reducing ROS levels, restoring the Bax/Bcl2 balance, and inhibiting apoptosis. Notably, all these alterations occur at a subclinical stage, characterized by normal echocardiographic function and the absence of noticeable fibrosis.

Our echocardiography results demonstrated that axitinib had no significant effect on the cardiac systolic or diastolic functions of C57BL/6 mice but did increase serum cTnT concentrations. These findings indicate that axitinib induces cardiac injury while global function remains normal, suggesting that cTnT release precedes functional decompensation. As a highly specific and sensitive circulating biomarker for cardiotoxicity [22], cTnT elevation explicitly indicates the disruption of cardiomyocyte membrane integrity or cell death, including apoptosis [23, 24]. These findings confirm that axitinib indeed causes damage at the cellular level. However, this early injury may be offset by the heart's compensatory capacity. The mice are at a stage where damage exists but has not yet led to detectable global functional failure—a stage of subclinical cardiac injury. This is similar to subclinical cardiac injury in clinical patients, which occurs in the early stages of myocardial damage. It is characterized by structural or cellular changes (such as myocardial fibrosis, inflammation, or cell injury) without overt symptoms (such as chest pain or dyspnea), and global cardiac dysfunction is difficult to detect using conventional methods (e.g., normal left ventricular ejection fraction on standard echocardiography) [25]. The underlying reason is that evidence of injury at the cellular and molecular levels (cTnT, apoptosis staining, and Bax/Bcl2) is more sensitive and can detect damage earlier and more directly. In contrast, echocardiography reflects global functional output at the organ level. During the early, mild, or focal stages of injury, changes in echocardiographic parameters may lag or remain insignificant because of cardiac compensation and the limitations of the ultrasound technique itself. Another reason for this phenomenon may be the duration of drug administration. Prolonging the treatment time could induce more severe cardiac injury that exceeds compensatory limits, which might then be detected as systolic or diastolic dysfunction by echocardiography. This also highlights the importance of monitoring early subclinical injury.

HE staining revealed that axitinib induced structural damage to the mouse heart, and positive TUNEL staining confirmed that axitinib induced cardiomyocyte apoptosis. However, collagen deposition was not prominent. A likely reason is that the injury has not yet progressed to the fibrotic stage, indicating the existence of a window for early intervention. Similarly, a study using a zebrafish embryo model revealed that axitinib treatment caused pericardial edema, but at a concentration of 50 μM, it only induced this effect in 20% of the embryos, suggesting relatively mild cardiotoxicity. Compared with other VEGFR‐TKIs, axitinib presents a wider therapeutic window [26], suggesting that it has a potentially better safety profile for clinical application.

Our results show that luteolin effectively alleviates axitinib‐induced cardiomyocyte injury by regulating the Bax/Bcl2 ratio. To further evaluate its relative efficacy, we introduced captopril, a classic angiotensin‐converting enzyme inhibitor, as a positive control in our in vitro experiments. The results indicated that both luteolin and captopril significantly reversed the abnormal increase in the Bax/Bcl2 ratio induced by axitinib. These findings suggest that the protective effect of luteolin is comparable to that of established cardiovascular drugs in blocking the apoptotic cascade. This antiapoptotic mechanism is highly consistent with the effects of luteolin in other cardiac disease models. For example, in myocardial ischemia/reperfusion injury models, luteolin significantly decreased Bax protein levels and increased Bcl2 expression, thereby reducing cardiomyocyte apoptosis [15]. Additionally, another study reported that luteolin significantly attenuated metabolic syndrome‐induced cardiac injury in rats by inhibiting apoptosis, inflammation, and oxidative stress while promoting autophagy [27]. Together with our data, these findings suggest that luteolin is a potential natural compound with protective effects against cardiac injury mediated by various pathological factors, including targeted drug toxicity.

It is necessary to clarify the timeframe of the animal model and the drug dosages used in this study. In terms of the axitinib dose, 50 mg/kg was selected on the basis of previous in vivo pharmacological studies, as this dose effectively simulates clinically significant antitumour effects in rodents [28]. For the modeling period, a short‐term 1‐week administration regimen was adopted, which was limited mainly by the acute toxicity tolerance of the drug in the animal model. In our preliminary experiments and a published study on axitinib‐induced severe renal podocyte injury [20], we reported that after 1 week of continuous administration, the mice exhibited obvious systemic deterioration, including weight loss and lethargy. Prolonging the administration period resulted in a high mortality rate. The rapid deterioration of the overall condition of the mice prompted us to further investigate whether they developed concurrent acute cardiac lesions. In terms of clinical relevance, although chronic heart failure is more common after long‐term targeted therapy, adverse cardiovascular events (such as early‐onset hypertension and acute cardiac decline) are equally important in clinical practice. As reported in clinical cases, some patients experienced reduced left ventricular ejection fraction and apical wall motion abnormalities just 6 days after receiving axitinib treatment [5]. Therefore, our 1‐week model effectively simulates the early/acute subclinical cardiac injury induced by axitinib in the clinic (characterized by elevated cTnT and apoptosis without a significant decrease in EF/FS), highlighting the importance of monitoring cardiac biomarkers during the early stage of targeted therapy. Furthermore, the luteolin dose (100 mg/kg) was chosen on the basis of previous pharmacological literature. This dose effectively exerts antioxidant and multiorgan protective effects in vivo without causing obvious side effects [20].

Nevertheless, it should be acknowledged that this 1‐week, high‐dose regimen represents an acute toxicity model that may not fully recapitulate the chronic, progressive cardiotoxicity observed in patients receiving long‐term axitinib therapy. Moreover, the systemic deterioration inherent to this acute schedule, while clinically informative for early events, may partially confound cardiac‐specific readouts. Therefore, future studies employing longer‐term, lower‐dose axitinib administration with pharmacokinetic monitoring to approximate clinical steady‐state exposure are warranted to validate whether luteolin's cardioprotective effects extend to the chronic setting.

Furthermore, it should be acknowledged that the axitinib concentration employed in our in vitro experiments (5 μM) exceeds the steady‐state plasma levels typically reported in patients. This concentration was rationally selected based on our 24‐h cytotoxicity assay, which yielded an IC_50_ of 10.47 μM in AC16 cells; the half‐IC_50_ dose was chosen to provide an optimal “pathological window”‐inducing clear apoptotic and oxidative stress responses while avoiding overwhelming necrosis that would obscure a potential protective effect. In standard 2D cell culture, the absence of systemic hemodynamic stress, neurohumoral factors, and the secondary cardiac insults that occur in vivo necessitates higher drug concentrations to acutely recapitulate the cumulative cellular injury observed during chronic clinical exposure. It is also possible that the immortalized AC16 line is inherently more drug‐tolerant than primary human cardiomyocytes. Notably, luteolin exhibited robust cytoprotection even against this exaggerated acute stress. However, we must acknowledge that its protective efficacy has not been directly tested at clinically achievable axitinib concentrations in the current study. When interpreted together with our in vivo data, these findings encourage, but do not yet confirm, the translational potential of luteolin at clinical doses. Future studies employing prolonged exposure protocols at lower, clinically relevant axitinib concentrations, preferably in primary human cardiomyocytes or engineered cardiac constructs, are therefore necessary.

Although the present study convincingly demonstrates that luteolin mitigates axitinib‐induced cardiotoxicity by modulating the ROS/Bax/Bcl2 axis, it is important to acknowledge that cardiovascular complications associated with TKIs typically involve a complex, multi‐pathway network. As reported in the literature, TKIs can also induce cardiomyocyte injury by triggering mitochondrial dysfunction [29], disrupting intracellular Ca^2+^ homeostasis leading to Ca^2+^ overload [30], and inhibiting the cardioprotective PI3K/AKT survival signaling pathway [31]. In fact, excessive ROS accumulation is frequently intertwined with abnormal mitochondrial permeability transition pore opening and Ca^2+^ mishandling, forming a vicious cycle of cellular stress [32, 33]. Furthermore, it is plausible that axitinib may suppress PI3K/AKT phosphorylation in cardiomyocytes through off‐target effects. Given the pleiotropic nature of luteolin as a multi‐target flavonoid [34], it remains to be explored whether its cardioprotective effects are partially mediated through these alternative pathways, such as preserving mitochondrial function or restoring PI3K/AKT signaling. Future studies employing pathway‐specific inhibitors and functional assessments at clinically relevant concentrations will be necessary to comprehensively elucidate the multidimensional molecular network underlying luteolin's protection against axitinib‐induced cardiotoxicity.

## 5 Conclusion

In summary, this study shows that axitinib induces subclinical cardiac injury in the early stage of administration. The mechanism is closely related to the imbalance of the Bax/Bcl2 ratio and cardiomyocyte apoptosis caused by abnormal ROS accumulation (Figure 9). This finding suggests that there is a window period in axitinib‐induced cardiac injury where pathological changes occur without obvious abnormalities in cardiac function. Therefore, closely monitoring cardiotoxicity biomarkers in the early stage of clinical targeted therapy is important for preventing severe cardiac decline later. Meanwhile, this study confirms that the natural compound luteolin effectively reverses the Bax/Bcl2 imbalance, showing a cardioprotective effect comparable to that of classic cardiovascular drugs. These results provide experimental evidence for understanding the cardiotoxic mechanisms of targeted drugs and suggest that luteolin is a potential candidate compound for preventing and treating such adverse drug reactions.

**Figure 9:**
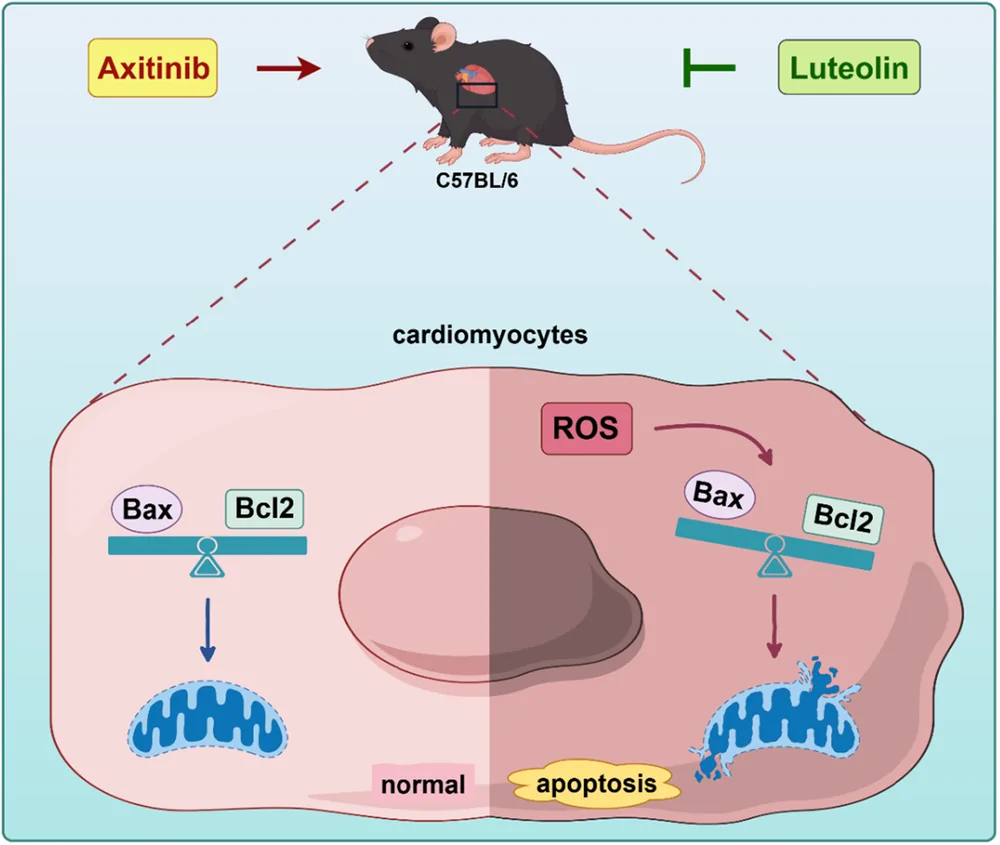
Luteolin alleviates axitinib‐induced cardiomyocyte apoptosis in C57BL/6 mice. Image by Figdraw.

## Author Contributions

**Si‐Wen Ji:** Investigation, Formal Analysis, Writing – original draft, Funding acquisition. **Xu Li:** investigation, formal analysis, writing – original draft. **Dong Tang:** investigation, formal analysis. **Lin Bai:** visualization. **Chun‐Lei Yu:** supervision, funding acquisition. **Na Niu:** conceptualization, writing – review and editing, funding acquisition. Na Niu and Chun‐Lei Yu are co‐corresponding authors; all authors approved the final version of the manuscript.

## Conflicts of Interest

The authors declare no conflicts of interest.

## Data Availability

The data that support the findings of this study are available from the corresponding author upon reasonable request.
